# Biofilm Formation and the Role of Efflux Pumps in ESKAPE Pathogens

**DOI:** 10.3390/microorganisms13081816

**Published:** 2025-08-04

**Authors:** Trent R. Sorenson, Kira M. Zack, Suresh G. Joshi

**Affiliations:** 1 Center for Surgical Infections & Biofilm, School of Biomedical Engineering, Science & Health Systems, Drexel University, Philadelphia, PA 19104, USA; trs336@drexel.edu; 2 Center for Surgical Infections & Biofilm, Department of Surgery, College of Medicine, Drexel University, Philadelphia, PA 19102, USA; kmz58@drexel.edu; 3 Center for Plasma in Health & Biomedical Engineering, Nyheim Plasma Institute, Drexel University, Philadelphia, PA 19104, USA

**Keywords:** *Acinetobacter baumannii*, biofilm, efflux pump, EPI, ESKAPE, MDR, multidrug resistance, nosocomial infection, surgical site infection, SSI

## Abstract

Nosocomial infections caused by ESKAPE pathogens represent a significant burden to global health. These pathogens may exhibit multidrug resistance (MDR) mechanisms, of which mechanisms such as efflux pumps and biofilm formation are gaining significant importance. Multidrug resistance mechanisms in ESKAPE pathogens have led to an increase in the effective costs in health care and a higher risk of mortality in hospitalized patients. These pathogens utilize antimicrobial efflux pump mechanisms and bacterial biofilm-forming capabilities to escape the bactericidal action of antimicrobials. ESKAPE bacteria forming colonies demonstrate increased expression of efflux pump-encoding genes. Efflux pumps not only expel antimicrobial agents but also contribute to biofilm formation by bacteria through (1) transport of molecules and transcription factors involved in biofilm quorum sensing, (2) bacterial fimbriae structure transport for biofilm adhesion to surfaces, and (3) regulation of a transmembrane gradient to survive the difficult conditions of biofilm microenvironments. The synergistic role of these mechanisms complicates treatment outcomes. Given the mechanistic link between biofilms and efflux pumps, therapeutic strategies should focus on targeting anti-biofilm mechanisms alongside efflux pump inactivation with efflux pump inhibitors. This review explores the molecular interplay between efflux pumps and biofilm formation, emphasizing potential therapeutic strategies such as efflux pump inhibitors (EPIs) and biofilm-targeting agents.

## 1. Introduction

Nosocomial infections remain a leading cause of complications, hospitalizations, morbidity, and death for individuals receiving surgery around the world. Although there have been advances made in controlling nosocomial infections by improving sterilization processes, operating room ventilation, and the use of antimicrobial agents, they reportedly account for 20% of all healthcare-associated infections [1]. Nosocomial infections are shown to lead to a two-to-eleven-fold increase in the risk of mortality for post-operative patients [2] and affect either the incision or the deep surgical sites of an operation [3]. The annual cost of Surgical Site Infections specifically is estimated at USD 3.3 billion and is considered the costliest healthcare-associated infection in the United States [4]. These infections can lead to an average increase of USD 20,000 in a patient’s hospital bill. Given the financial and detrimental impact of nosocomial infections on patient health, it is imperative to understand molecular mechanisms for nosocomial infections and their pathogenesis. Currently, there are only fifth-generation antibiotics available to treat the increasing burden of antibiotic-resistant bacteria [2]. Therefore, there is significant importance in developing novel antibiotics to target key pathogenic steps in nosocomial bacterial colonization. A better understanding of the relationship between efflux pump mechanisms and their impact on biofilm formation is important for a better understanding of potential drug targets for interfering with steps in pathogenesis.

Most nosocomial infections are caused by “ESKAPE” pathogens, which have acquired resistance to many of the commonly used antibiotics [5]. The Center for Disease Control and Prevention (CDC), the National Institute of Health (NIH), the US Department of Defense (DOD), and the World Health Organization (WHO) have listed ESKAPE pathogens as high-priority, immediate-attention pathogens that are likely to contribute to major dissemination of resistance. The term “ESKAPE” includes six bacteria which have developed multidrug resistance (MDR): *Enterococcus faecium*, *Staphylococcus aureus*, *Klebsiella pneumoniae*, *Acinetobacter baumannii*, *Pseudomonas aeruginosa*, and *Enterobacter* spp. [6]. MDR in ESKAPE pathogens is a leading threat to global health and often results from the overprescription of antibiotics, incorrect antimicrobial usage, and lack of bactericidal antibiotics for these pathogens [7]. Understanding the mechanisms of multidrug resistance and the development of antimicrobials which escape these mechanisms of resistance remains essential in combating the global threat of ESKAPE pathogens. Most antibiotic resistance genes are carried on plasmids, bacterial chromosomes, or transposons [8]. Drug alteration/inactivation, drug receptor modification or blockage, efflux pump activation resulting in reduced intracellular drug accumulation, and biofilm formation are leading causes of antimicrobial resistance mechanisms [9]. For example, isolates of *A. baumannii* produce carbapenemases to inactivate carbapenem antimicrobials and produce antibiotic-impenetrable biofilms as part of their resistant repertoire [10]. In addition, the quinolone transporter NorA from methicillin-resistant *S. aureus* (MRSA) is upregulated during infection to remove norfloxacin inhibiting their bactericidal potential [11].

Previous research has indicated that the overexpression of efflux pumps is a leading cause of multidrug resistance in hospital-based infections [1,10]. Efflux pumps are a large class of transporters aimed at removing harmful substances and metabolites and assisting in the uptake of nutrients for bacterial growth in the presence of many antibiotics [11,12]. For instance, a review by Prajapati et al. highlights the effectiveness of outer membrane porins in the efflux pumps of Gram-negative efflux for the uptake of essential nutrients for bacterial survival [13]. Efflux pumps lead to MDR due to their ability to export antimicrobial drugs given for bacterial infection treatment into the extracellular environment. In addition, efflux pumps have been shown to regulate nutrient and heavy metal levels and assist with the extrusion of bile and other toxic substances, allowing pathogens to flourish and grow without the threat of lethal substances [14,15]. Most of these efflux pumps function through either primary or secondary mechanisms. Primary efflux pumps harness energy through the hydrolysis of ATP to drive the transport of substances across the microbial membrane. Secondary efflux pumps establish a proton gradient and utilize this gradient to assist with the transport of antimicrobial substances. Some of the efflux pumps, such as ABC transporters, are directly driven by the energy of ATP hydrolysis, regardless of the presence or absence of a proton gradient. The efflux pumps of Gram-negative bacteria can be grouped into five major superfamilies: (1) ATP-binding cassette (ABC), a primary efflux pump, (2) small multidrug resistance family, (3) multidrug and toxin extrusion (MATE) family, (4) major facilitator superfamily (MFS), and (5) resistance nodulation cell division family (RND), which are all secondary efflux pump transporters [13,16]. Biofilms are essential for the pathogenesis of nosocomial infections. Biofilms are complex multicellular communities of microorganisms that produce extracellular matrices to facilitate attachment to biotic and abiotic surfaces [17]. Moreover, their role in multidrug resistance mechanisms such as efflux pumps has been shown to increase bacterial survival in harsh toxic environments and contribute to biofilm formation [18].

Biofilm formation on the surface has been shown to correlate with the pathogenesis of Surgical Site Infections (SSIs) [17,19]. The polysaccharide-rich extracellular environment makes it hard for antimicrobial compounds to penetrate and reach the pathogen [20]. Biofilms are colonies of microorganisms which utilize extracellular substances to attach and adhere to biotic and abiotic surfaces allowing for microbial colonization and biofilm-producing cells to remain stationary [21]. With enhanced biofilm-forming capacity, the pathogens acquire genetic diversity and survive in diverse environments while colonizing many different surfaces [17]. The extracellular matrix associated with biofilm-producing microbial colonies contain polysaccharides, nucleic acids, lipids, and proteins. The polysaccharides in the biofilm’s extracellular layer allow for easy attachment to innate surfaces and formation of a complex three-dimensional network, which allows for the rapid growth of bacterial-forming colonies [22]. The life cycle of biofilm formation includes (1) attachment, (2) colonization, (3) development, (4) maturation, and finally (5) active dispersal [23], where microorganisms migrate and colonize additional surfaces. In addition, biofilm-forming colonies also assist with resistance to environmental and physiological stressors which impede the survival of these pathogens [24,25]. The biofilm serves as a protective barrier and insulation from these stressors and harsh environmental conditions.

Previous research with beta-lactam antibiotics demonstrates the upregulation of AdeFGH efflux pumps in biofilm-forming *A. baumannii* isolates [26]. A study by He et al. in 2015 used three strains of *A. baumannii* to evaluate antimicrobial susceptibility and gene expression involved in biofilm expression [27]. Upregulation of efflux pump genes *adeG* and *abaI* correlated with biofilm formation in colonies exposed to levofloxacin and meropenem [27]. Similarly, research has shown that carbapenem-susceptible strains of *A. baumannii* upregulate the expression of efflux pumps to survive the pressure of antimicrobials [28]. The overproduction of bacterial efflux pumps plays a direct role in reducing the susceptibility of carbapenem and other antibiotics in *A. baumannii* pathogenesis. The evident connections between efflux pump function and biofilm formation indicate a potential connection for the development of MDR therapeutics. MDR resistance-related deaths are set to reach ten million annually by 2050 [29]. Developing therapeutic strategies to target efflux pumps and biofilm formation remains essential to lessen the global burden of antibiotic resistance. The purpose of this review is to establish mechanistic and gene expression connections between efflux pumps and biofilm-producing microbial colonies in ESKAPE pathogens. Elucidating these connections may provide additional therapeutic insights to impede the global burden of MDR nosocomial bacterial infections.

## 2. Efflux Pump Genes and Biofilm-Forming Capacity

Research has shown associations between efflux pump genes and biofilm-producing colonies of ESKAPE pathogens. In *P. aeruginosa* isolates, RND and MFS efflux pump genes demonstrate increased expression in biofilm-producing strains [30]. RND efflux pumps form a tripartite complex with proteins on the outer membrane, a complex in the periplasmic space, as well as an additional protein complex in the inner membrane space. This allows for direct movement of substances from the inside of the cell to the extracellular environment. Similarly, the MFS efflux pump is the largest group of secondary efflux pump transporters; however, the inner membrane protein does not extend into the periplasm [27,29,31]. Instead, there is an adaptor protein which functions to connect the periplasmic protein complex to the inner membrane protein complex [27]. The transmembrane protein complex of RND efflux pumps functions to assist with the transport of nutrients and additional toxic materials out of the cell during biofilm formation. This allows essential nutrients for colony survival to efficiently complete the development and maturation phases of biofilm development.

Similarly, Ugwuanyi et al. (2021) found that one hundred percent of biofilm-producing *P. aeruginosa* colonies expressed *mexA* genes and above ninety percent of the same colonies expressed *mexB* and *oprM* genes [32] The MexAB-OprM efflux pump belongs to the RND family of efflux pumps and has been shown to be overexpressed in *P. aeruginosa* MDR isolates [33,34]. Alav et al. (2018) describe the role of the MexAB-OprM efflux pump in the regulation of the dispersal of quorum sensing (QS) in bacterial-forming colonies [25]. This mechanistic link will be discussed later.

In *E. faecalis*, the chromosomally encoded ABC-type efflux pump EfrAB (from the *efrA*/*efrB* genes) and the MFS-type transporter EmeA are frequently associated with decreased susceptibility to multiple antibiotics and antiseptics. Surveys have found efrAB present in up to 100% of clinical *E. faecalis* isolates and closely linked with lower susceptibility to chlorhezidine [35]. Transcriptomic studies likewise show that genes encoding efrEF (another ABC transporter regulated by *chlR*) are among the most highly upregulated during chlorhexidine exposure, suggesting a biofilm-relevant stress induction [35]. More broadly, reviews of efflux pumps in bacterial biofilm formation describe that in many bacteria, pump gene expression increases in the biofilm phase, and these pumps contribute to biofilm persistence by exporting quorum-sensing molecules, EPS components, and waste products, thereby contributing to the biofilm’s antimicrobial tolerance [36].

In another ESKAPE member, *Enterobacter* spp. (e.g., *E. cloacae*, *E. aerogenes*), multidrug resistance is strongly associated with overexpression of RND-family pumps such as AcrAB-TolC and plasmid-borne systems like EefABC. Resistant clinical isolates frequently display elevated AcrAB levels often regulated by activators like RamA or MarA, with corresponding resistance to macrolides, quinolones, and tetracyclines [37]. While direct studies in Enterobacter biofilms are fewer, the RND family’s broad role in biofilm formation, including efflux of quorum-sensing autoinducers, modulation of motility, and EPS secretion, is well-documented across Gram-negative bacteria [38].

In addition, in biofilm-producing isolates of *A. baumannii*, efflux pump genes *adeB*, *adeG*, and *adeJ* and outer membrane protein genes *carO* and *ompA* were overexpressed [26]. The AdeFGH efflux pump has the most significantly increased gene expression in biofilm-producing *A. baumannii* [27]. The AdeFGH efflux pump belongs to the RND efflux pump family and has been shown to be overexpressed in MDR *A. baumannii* isolates [10,16]. Similar to the findings of Alav et al., 2018 [25], the *A. baumannii* isolates also demonstrated overexpression of the autoinducer abaI when transitioning from planktonic to biofilm-producing colonies [26]. This suggests a link between quorum sensing’s role in bacterial biofilm production. Similarly, adeIJK, adeABC, and adeFGH efflux pump mutants of *A. baumannii* demonstrated a decrease in biofilm-forming capacity [39]. A deletion of these efflux pump-encoding genes diminished biofilm formation in *A. baumannii*, indicating a clear role for Ade efflux pumps in *A. baumannii* biofilm formation.

Outer membrane proteins OmpC, OmpF, and OmpT showed significantly increased expression in *E. coli* biofilm-producing colonies [32]. *Omp* encodes for the outer membrane protein of the RND efflux pump in *E. coli* [40]. Increased expression of the outer membrane protein in *E. coli* regulates the movement of nutrients to the extracellular environment for the formation of the polysaccharide- and protein-rich biofilm layer surrounding the aggregates of bacteria. There is immense demand for nutrients in the attachment and colonization stages of biofilm development and these outer membrane proteins play a role in nutrient availability to the microorganism. Previous research also demonstrates the upregulation of these outer membrane proteins in the attachment of *E. coli* and *A. baumannii* to abiotic surfaces [39,41,42]. There is a direct role of these outer membrane proteins in the initiation of biofilm formation and attachment. Similarly, the AcrAB-TolC RND-based tripartite efflux pump demonstrated increased expression during biofilm formation after exposure to many antibiotics [43]. Specifically, the TolC protein demonstrated overexpression [43], which includes the outer membrane component of the tripartite efflux pump [44]. Through gene association studies, researchers have demonstrated a clear link between efflux pump gene overexpression in biofilm-producing colonies [45]. A mechanistic link exists between efflux pumps and bacterial biofilm-forming capacity. Table 1 summarizes the efflux pump gene expression in biofilm-producing bacterial colonies. Given the direct role of biofilm formation and overexpression of efflux pumps, we suggest that future therapeutic strategies could work to inhibit efflux pumps and limit biofilm pathogenies. The pathogenesis of many of the nosocomial infections relies on the formation of biofilms for pathogenies. Efflux pump inhibitors could serve as potential future antibiotics to limit the biofilm spread of nosocomial infections.

## 3. Mechanistic Link Between Efflux Pump and Biofilm Formation

### 3.1. Quorum-Sensing Molecules

Biofilm-forming cells communicate with each other through a process called quorum sensing [48]. The *abaI* gene encodes a protein required for the signaling process of quorum sensing in *A. baumannii* biofilm formation [49]. Gene expression analysis of biofilm-producing colonies of *A. baumannii* shows overexpression of the *adeG* gene of the AdeFGH efflux pump and the *abaI* gene [26]. AbaI gene upregulation assists with the transformation of planktonic bacteria to biofilm-producing cells, suggesting a potential role of the efflux pump function and biofilm formation. He et al. (2015) [27] explain how AdeFGH efflux pumps play a role in the cotransport of acylated homoserine lactones (AHLs) along with antibiotics during biofilm formation. There is an increase in the concentration of AHL molecules in the intracellular environment which leads to the formation of abaR-AHL complexes which induce the expression of AbaI during quorum sensing regulation. The *AbaR* gene encodes molecules important for the signaling involved in bacterial quorum sensing [50]. In particular, *abaR* encodes an autoinducer synthase molecule essential for the signaling involved in sharing information about cell density and regulating gene expression in biofilm formation [50]. AbaR functions to assess the microenvironment of bacterial colonies to continue the biofilm-forming cascade and upregulate *AbaI* gene expression. The AHLs are transported to the extracellular environment via the AdeFHG efflux pumps [27]. AHLs interact with *A. baumannii* cells and enhance cell-cell interactions through the upregulation of target genes for bacterial elastin and virulence factors for biofilm formation [51]. Deletion of AbaI decreased biofilm formation ability in *A. baumannii* [46] demonstrating its importance and role in pathogenesis. In addition, the RND family of efflux pumps has been shown to transport fatty acids, QS molecules, and QS precursors [18] which allow communication in biofilm-producing bacterial colonies in Gram-negative bacteria. Much of the current drug development research is looking to develop quorum quenching (QQ) mechanisms to target the inhibition of biofilm-forming mechanisms.

**Figure 1:**
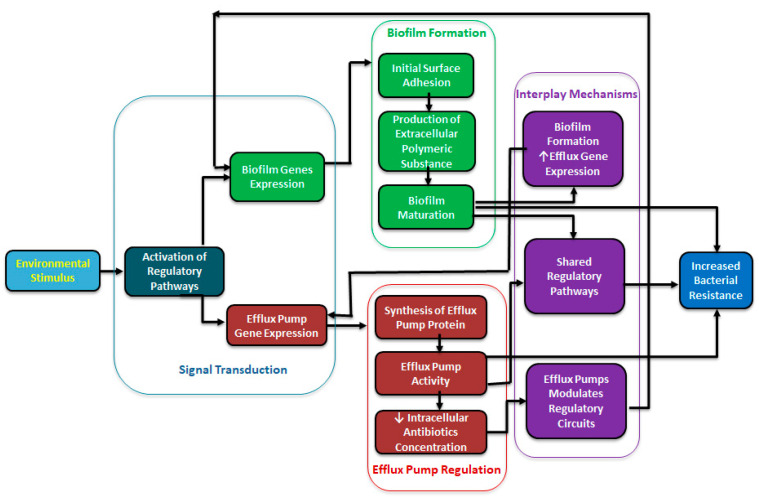
A schematic diagram showing a regulatory interplay between efflux pump and biofilm formation.

Similarly, a BPE-ompR efflux pump inactivation in *P. aeruginosa* shows downregulation of a lecA:lux quorum sensing molecule regulation by inhibiting the transport of these molecules [45]. In addition, *Salmonella enterica* Typhimurium treated with the AcrAB-TolC RND efflux pump inhibitor Phenylalanine-Arginine Beta-Naphthylamide (PAβN) also blocked the transport of AHL quorum sensing molecules [52]. The inhibitor studies (using PAβN or 1-(1-Naphthylmethyl)-piperazine (NMP) efflux pump inhibitors) have also shown significant reductions in biofilm formation in *E. coli*, *K. pneumoniae*, and *P. aeruginosa*, implying that efflux activity directly supports biofilm integrity and maturation [53]. Figure 1 shows a schematic interplay between the efflux pumps and biofilm formation and increased antimicrobial resistance. Efflux pumps transport essential signaling molecules for biofilm formation and modulate signaling and influence the physical-chemical properties of the extracellular matrix and metabolic variation [54]. Therapeutic targets may benefit from utilizing efflux pump inhibitors (EPIs) to trap the movement of these quorum-sensing molecules with the hopes of inhibiting bacterial biofilm formation.

### 3.2. Fimbriae and Bacterial Mobility

Bacterial biofilm formation uses chaperone-usher pathways (cup) fimbriae to secure attachment to abiotic and biotic surfaces in *P. aeruginosa*. *P. aeruginosa* produces four types of non-flagellar surface filaments used in pilus assembly, which is referred to as the chaperone-usher pathway [55]. Chaperone-usher proteins are used in the export of fimbrial protein structures to the extracellular environment allowing for initial attachments of bacterial biofilms. The chaperone proteins deliver a pillin-chaperone complex which allows for the fibril assembly when they reach the extracellular environment. CupA-C relates to initial fimbriae assembly in the initial stages of biofilm formation. Two genes (*cupB* and *cupC*) are controlled by a two-component (RocA1-RocR) system, where RocA1 upregulates the expression of CupB and CupC, and RocR downregulates the two genes [50,51,56,57,58].

The Roc system is a two-component system which functions to allow bacteria to deliver signals to adapt and respond to diverse environments. These two-component systems play a role in the regulation of virulence factors in *P. aeruginosa* pathogenesis [59]. In a particular study observing bacterial *P. aeruginosa* isolates from the lungs of cystic fibrosis patients, there was upregulation of RocA1 protein leading to CupC expression in a two-component system [50]. However, the same elements controlling upregulation of RocA1 demonstrated a decrease in *mexAB-oprM* and *mexR* gene expression [50] belonging to the RND efflux pump family. The downregulation of mexAB-oprM RND efflux pumps through RocA1 demonstrates a need for fimbria structures in early biofilm adhesion to surfaces in *P. aeruginosa*. These results indicate that *P. aeruginosa* in CF patients appears to rely more on initial biofilm attachment and assembly rather than efflux pump expression in multidrug resistance. Targeting biofilm attachment and fimbriae assembly through regulating cup fimbriae might serve as a promising therapy to eradicate the ability of *P. aeruginosa* to form bacterial biofilms.

### 3.3. Efflux Pumps and the Ionic-Transmembrane Gradient

The upregulation of ATP-driven efflux pumps during biofilm formation proves an important mechanistic connection between regulating the biofilm microenvironment while transporting bactericidal antibiotics into the extracellular environment. ATP-driven efflux pumps function by utilizing a transmembrane gradient largely through the influx of H+ protons through an antiporter exchanger [60]. When the established proton gradient is created, an ATP synthase molecule can drive ATP formation. The generated ATP molecule is used by the ATP-driven efflux pump to remove the antimicrobial drugs from the intracellular environment. Upon removal of the bactericidal agents, the bacteria now have the nutrients and the environment to begin biofilm attachment and aggregation [55].

The function of the antiporters, ATP synthase molecules, and the ATP-driven efflux pumps is to protect the bacterial cells from the potential lysis in hyperosmotic environments [55]. Cells suspended in hyperosmotic solutions are at risk of lysis, given that their membranes are permeable to water and specific ions. Transmembrane transporters function to prevent a significant osmotic difference between the extracellular and intracellular environments, preventing the risk of bacterial lysis. In addition, the ability to withstand positive osmotic pressure gradients allows these bacteria to handle a wide range of environments when establishing biofilm colonies [55]. Biofilm formation requires immense adaptability to nutrient-poor and diverse environments. The function of this diverse range of antiporters, ATP synthase molecules, and efflux pumps may serve to allow these biofilm-producing colonies to adapt to changing environments and ensure the bacteria have the nutrients and ability to thrive when establishing biofilms.

## 4. Efflux Pump Inhibitors and Biofilm Formation

Efflux pump inhibitors (EPIs) work to inhibit the function of the efflux of antimicrobial substances across the bacteria. The EPIs are a potential solution in combating many of the resistant strains of Gram-negative bacteria; however, none are clinically approved due to the many requirements needed to make them successful [18]. Specifically, Phenylalanine-arginine-β-napthylamide (PaβN) is a synthetically derived EPI, widely considered the broadest spectrum of efflux pumps [61]. The mechanism of action of PaβN is unknown; however, it is thought to work as a competitive inhibitor of the efflux pump antimicrobial binding site or by altering bacterial cell permeability [62]. The addition of PaβN and 1-(napthylmethyl)-piperazine (NMP), a synthetically derived noncompetitive efflux pump inhibitor [59], demonstrated decreased biofilm-forming capacity in *E. coli* and *S. aureus* [63]. EPI’s chlorpromazine, an antipsychotic used as an efflux pump inhibitor, and carbonyl cyanide 3-chlorophenylhydrazone (CCCP) demonstrated decreased biofilm formation in *E. coli* and *K. pneumoniae* [63,64]. Given the role of biofilm inhibition when treated with EPIs, there is a clear mechanistic link between multidrug efflux pumps and biofilm formation. Given the potential role of PaβN as an agent decreasing cell membrane permeability, it may indicate that PaβN plays a role in destroying the osmotic pressure gradient necessary for establishing bacterial biofilms in diverse environments. There remains immense potential in utilizing EPIs to not only combat MDR but also assist in eradicating the threat of Gram-negative bacterial biofilms.

In addition, *S. enterica* Typhimurium demonstrated decreased biofilm-forming capacities when treated with dual PaβN and antibiotics norfloxacin and ciprofloxacin [52]. The fitness level of *S. enterica* Typhimurium after dual treatment demonstrated decreased bacterial motility and flagella movement, indicating a potential role for flagella function in biofilm formation. In addition, Dawan et al. (2022) indicated that PaβN blocked the transport of AHLs, leading to a reduction in available AHLs to assist in quorum sensing and bacterial congregation [61]. The dual action of EPI as quorum quenchers and biofilm inhibitors indicates the dual role of efflux pumps in multidrug resistance and biofilm formation. The multifactor nature of these EPIs demonstrates immense promise for future drug development in EPI and controlling biofilm development.

In addition, treatment with a NorA efflux pump inhibitor in *S. aureus* demonstrated a significant decrease in biofilm mass and biofilm-forming capacity [65]. *P. aeruginosa* strains demonstrated that PaβN in combination with the iron chelator EDTA showed a decreased biofilm-forming capacity [66]. With iron as an essential component for biofilm growth and attachment [21], inactivating a substrate for biofilm formation and the use of an EPI demonstrates therapeutic promise in combating MDR.

Reserpine, typically used to treat high blood pressure, is a plant-derived alkaloid EPI that directly binds to MFS and RND efflux pumps in Gram-negative bacteria [67] and has been shown to increase the susceptibility of *A. baumannii* clinical isolates to levofloxacin [68]. Reserpine has also been demonstrated as a potent biofilm inhibitor in *K. pnuemoniae* [69]. With reserpine’s demonstrated role as an alkaloid EPI, reserpine also impacts the ability of *K. pnuemoniae* to form new biofilms, further elucidating the role of efflux pumps in biofilm formation. There remains great promise in using combination therapies of EPI and biofilm inhibition to target the MDR seen in ESKAPE Gram-negative bacteria. Table 2 shows the representative EPIs and their impact on biofilm formation.

## 5. Biofilm Inhibition as MDR Therapy

Efflux pump inhibitors demonstrate a clear impact on bacterial biofilm formation; however, research has also focused on direct biofilm inhibitors to decrease biofilm pathogenicity and fitness. Quorum quenching (QQ) mechanisms work to directly inhibit the quorum sensing necessary to propagate bacterial communication and signaling for biofilm formation. The QS system can interfere in multiple ways: (1) decreased expression of the sensing molecules, (2) enzymatic degradation of signaling molecules, (3) competitive inhibitors for QS molecule receptor binding, and (4) inhibition of QS molecule gene expression [74]. For example, through the enzymatic destruction of quorum-sensing AHL molecules, there could be an inhibition in bacterial sensing and a decrease in biofilm formation [46]. Mayer et al. (2020) found that the combined use of the QQ enzyme Ai20J, an AHL-degrading enzyme, and DNase reduced bacterial biofilm-forming capacity in strains of *A. baumannii* [75] . Nucleic acids, proteins, and carbohydrates are a large component of the bacterial cell’s extracellular matrix in biofilms, indicating that the addition of DNase plays a role in degrading this necessary component. In addition, MomL, an AHL-degrading enzyme, demonstrated decreased biofilm formation and increased bactericidal capability of antibiotics in *A. baumannii* [72,76]. Similarly, the use of palmitoleic acid (POA) and myristic acid (MOA) demonstrated decreased biofilm formation in *A. baumannii* through a reduction in AHL signaling molecules. The monounsaturated fatty acids decreased AbaR expression needed for the signaling pathway for the expression of AHLs and demonstrated a decrease in *A. baumannii* motility [77]. Interestingly, the use of erythromycin demonstrated biofilm inhibitory effects on *A. baumannii* and *P. aeruginosa* by inhibiting the QS pathway. The use of erythromycin likely impacts the synthesis of AHL molecules involved in QS by inhibiting the AbaI autoinducer synthase [70,78]. In addition, erythromycin may directly inhibit signal reception of AHL inhibiting quorum sensing activity [79].

Bacterial motility and aggregation are key fitness factors influencing successful bacterial biofilm formation. Nicol et al. reported that their use of POA and MOA reduced bacterial mobility and biofilm dispersing capacity in *A. baumannii* [70,77]. In addition, linalool, a terpene alcohol found in many essential oils, disrupted the aggregation and adhesion of *A. baumannii* biofilm colonies [73]. Similarly, cathelicidin, a natural human antimicrobial peptide, inhibited bacterial motility and swimming in *P. aeruginosa* [80]. There remains enormous potential in inhibiting successful biofilm formation as a method for decreasing bacterial fitness and biofilm-forming capacity.

Combining efflux pump inhibition and biofilm inhibition therapies in Gram-negative multidrug-resistant organisms may demonstrate great promise in combating the disease burden of MDR bacterial strains. Photodynamic therapy utilizes specific wavelengths of light to generate reactive oxygen species (ROS) as an antimicrobial agent [81]. In methicillin-resistant *S. aureus* (MRSA), photodynamic therapy demonstrated reduced efflux pump expression and function as well as an ability to decrease biofilm-forming capacity in MRSA strains [81]. Photodynamic therapy limits the signaling molecules required in biofilm formation and therefore limits bacterial colonies’ ability to form colonies in their pathogenesis [82]. Targeting combined efflux pump inhibition and biofilm formation may prove beneficial in decreasing the relative fitness of Gram-negative nosocomial bacterial infections. Table 3 summarizes the biofilm inhibitors and their role in disrupting biofilm adhesion and fitness.

## 6. Conclusions

Efflux pumps remove many of the antibiotics from within cells to the extracellular environment contributing to increasing antibiotic resistance in pathogens causing nosocomial infections. In addition, there is increasing research from studies suggesting the role of efflux pumps in biofilm formation. Given the public health priorities and economic burden of healthcare-associated nosocomial infections, there remains a clear need for therapy targeting the MDR strains of bacteria. The presented literature and mechanisms indicate a link between efflux pumps and bacterial biofilm formation observed in ESKAPE pathogens. Known efflux pump inhibitors (EPIs) have been shown to limit bacterial biofilm formation. In addition, biofilm inhibitors have been extensively studied in Gram-negative pathogens, and their efficacy in targeting biofilm-forming capacity and aggregation remains evident. Therefore, combining efflux pump inhibition and biofilm inhibition therapies in such multidrug-resistant organisms may demonstrate a great approach in developing strategies to target the immense burden of nosocomial infections. Further research and understanding about the role of efflux pumps and their involvement in biofilm formation are important in taking newer approaches in the development of therapeutic agents to combat antibiotic resistance.

## Figures and Tables

**Table 1:** Overexpression of the efflux pump genes in biofilm-producing colonies of ESKAPE pathogens.

Biofilm-Producing Bacteria	Overexpressed Efflux Pump Gene	Efflux Pump Family	Reference
*P. aeruginosa*	*mexA*, *mexB*	RND type	Zahedani et al. [18]
	*tetA*, *tetR*	MFS-type	Ugwuanyi et al. [32]
	*oprM*	RND type	He X. et al. [27]
*A. baumannii*	*adeB*, *adeG*, *adeJ*	RND type	He X. et al. [27]
*E. coli*	*ompC*, *ompF*, *ompT*	RND type	Schembri et al. [43]
	*tolC*	RND type	Bailey et al. [46]
*E. faecalis*	*efrA*, *efrB*	RND type	Pereira et al. [35]
*Enterobacter* spp.	*AcrAB TolC*, *EefABC*	RND type	Li and Nikaido [37]
*K. pneumoniae*	*acrA*, *oqxA*	RND type	Tang et al. [47]
*K. pneumoniae*	*emrB*	SMR, MFS-type	Tang et al. [47]

**Table 2:** Efflux pump inhibitors (EPIs) demonstrated to reduce biofilm-forming capacity in ESKAPE pathogens.

Treatment	Biofilm-Reducing Bacterial Strains	Proposed Impact on Biofilm Formation	References
Specifically, Phenylalanine-arginine-β-napthylamide (PaβN) (competitive EPI)	*E. coli* *S. aureus*	(1) Destroys osmotic pressure gradient necessary for biofilm growth in diverse conditions (2) Blocks transport of essential AHLs in biofilm quorum sensing	Sidrim et al. [70]; Magesh et al. [71]
PaβN (EPI) + norfloxacin or ciprofloxacin (antibiotics)	*S. enterica* Typhimurium	Decreases bacterial motility and flagella movement	Baugh et al. [72]; Dawan et al. [61]
PaβN (EPI) + EDTA (iron chelator)	*P. aeruginosa*	Iron, an essential component of biofilm formation, combined with competitive EPI decreases bacterial relative fitness	Liu et al. [73]
1-(napthylmethyl)-piperazine (NMP) (noncompetitive EPI)	*E. coli* *S. aureus*	NA	Sidrim et al. [70]; Magesh et al. [71]
Chlorpromazine (antipsychotic EPI)	*E. coli* *K. pneumoniae*	NA	Sidrim et al. [70]
carbonyl cyanide 3-chlorophenylhydrazone (CCCP) (EPI)	*E. coli* *S. aureus* *K. pneumoniae*	NA Decreased biofilm formation	Sidrim et al. [70]; Magesh et al. [71] Tang et al. [47]
Reserpine (alkaloid EPI)	*K. pneumoniae*	Impacts *K. pneumoniae*’s new biofilm formation ability	Sidrim et al. [70]

**Table 3:** Biofilm-forming inhibitors and their mechanisms for decreasing ESKAPE pathogen biofilm fitness.

Treatment	Bacterial Strains	Mechanism	References
Ai20J	*A. baumannii*	AHL-degrading enzyme limiting quorum-sensing signaling	Mayer et al. [75]
MomL	*A. baumannii*	AHL-degrading enzyme limiting quorum-sensing signaling	Mayer et al. [75]
Palmitoleic Acid (POA)	*A. baumannii*	(1) Decrease abaR signaling needed for expression of AHLs in quorum sensing (2) Decrease in bacterial motility	Mayer et al. [75]; Nicol et al. [77]
Myristic Acid (MOA)	*A. baumannii*	(1) Decrease abaR signaling needed for expression of AHLs in quorum sensing (2) Decrease in bacterial motility	Mayer et al. [75]; Nicol et al. [77]
Erythromycin	*A. baumannii* *P. aeruginosa*	Inhibition of the quorum-sensing pathway	Mayer et al. [75];
Cathelicidin	*P. aeruginosa*	Decrease in bacterial motility	Zhang et al. [83]; De la Fuente-Nunez et al. [84]
Linalool	*A. baumannii*	Disrupted bacterial aggregation and adhesion	Alves et al. [85]; De la Fuente-Nunez et al. [84]
Photodynamic therapy	MRSA	(1) Decrease in efflux pump gene expression (2) Reduction in biofilm-forming ability	Yu et al. [82]

## Data Availability

No new data were created or analyzed in this study. Data sharing is not applicable to this article.
